# Factors influencing treatment adherence in paracoccidioidomycosis in Brazil

**DOI:** 10.1002/puh2.49

**Published:** 2023-01-11

**Authors:** Wdson Luis Lima Kruschewsky, Ricardo Dal Col Batista, Hosana Ewald Oliveira, Aloísio Falqueto, Paulo Mendes Peçanha, Tânia Regina Grão Velloso, Sarah Gonçalves Tavares, Creuza Rachel Vicente

**Affiliations:** ^1^ Faculdade de Medicina, Universidade Federal do Espírito Santo Vitória Brazil; ^2^ Programa de Pós‐Graduação em Doenças Infecciosas, Universidade Federal do Espírito Santo Vitória Brazil; ^3^ Departamento de Medicina Social, Universidade Federal do Espírito Santo Vitória Brazil; ^4^ Departamento de Clínica Médica, Universidade Federal do Espírito Santo Vitória Brazil; ^5^ Programa de Pós‐Graduação em Clínica Odontológica, Universidade Federal do Espírito Santo Vitória Brazil; ^6^ Departamento de Clínica Odontológica, Universidade Federal do Espírito Santo Vitória Brazil; ^7^ Departamento de Patologia, Universidade Federal do Espírito Santo Vitória Brazil

**Keywords:** alcohol drinking, Brazil, paracoccidioidomycosis, patient compliance, smoking, treatment adherence

## Abstract

**Introduction:**

Paracoccidioidomycosis is a systemic mycosis endemic to Latin America, with frequent reports of poor treatment adherence resulting in worse clinical outcomes. The present study evaluated factors related to treatment adherence in patients with paracoccidioidomycosis.

**Methods:**

This cross‐sectional study included data from medical records of patients with paracoccidioidomycosis treated between 2005 and 2018 in a reference hospital in southeastern Brazil. Clinical variables, demographic characteristics, and smoking and alcohol consumption were collected and analyzed.

**Results:**

Of the 198 cases included, most were adults (median age = 51 years), males (93.4%), presented chronic paracoccidioidomycosis (83.8%), reported smoking (90.4%) and alcohol consumption (84.8%), and 56.6% adhered to treatment. Treatment adherence was associated with the discontinuation of alcohol consumption (odds ratios (OR) = 5.227; 95% confidence interval (CI) = 2.697–10.129) and smoking (OR = 3.365; 95% CI = 1.781–6.358) during therapy.

**Conclusions:**

Patients' continued consumption of alcohol and tobacco was associated with non‐adherence to treatment, suggesting that a joint therapeutic approach that addresses alcohol and tobacco use alongside paracoccidioidomycosis treatment may hold promise for improving adherence and patients' quality of life.

## Graphical abstract

Non‐adherence to treatment is common among paracoccidioidomycosis patients. Continued consumption of alcohol and tobacco contributes to non‐adherence to treatment. A joint therapeutic approach that addresses alcohol and tobacco use alongside paracoccidioidomycosis treatment may hold a promise in improving adherence and patients' quality of life.

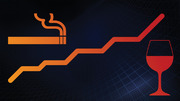

## INTRODUCTION

Paracoccidioidomycosis is a systemic mycosis endemic to Latin America caused by *Paracoccidioides brasiliensis* (S1a and S1b), *Paracoccidioides venezuelensis* (PS4), *Paracoccidioides restrepiensis* (PS3), *Paracoccidioides americana* (PS2), and *Paracoccidioides lutzii*. The disease can affect any organ and system, causing anatomical and functional changes due to the chronic granulomatous inflammation associated with fibromatosis [1, 2, 3]. Acute or subacute paracoccidioidomycosis occurs mainly in children, adolescents, and young adults. In contrast, chronic paracoccidioidomycosis is typical of adults older than 30, especially men, and is the most common presentation of the disease, representing 74%–96% of cases. Sequelae may affect the lungs, adrenal glands, larynx, central nervous system, and skin, leading to manifestations such as cough and chronic dyspnea, hypoxemia, hypotension, dysphonia, motor impairment, epilepsy, and microstomia [4].

The main drugs used to treat paracoccidioidomycosis are amphotericin B for severe and disseminated presentation and itraconazole or cotrimoxazole for mild and moderate cases or maintenance therapy of severe forms, with administration lasting between 9 and 24 months. Therapeutic adherence is essential for treatment success since the drugs used are effective, with no evidence of resistance [4, 5]. Prolonged drug therapy and regular patient monitoring during this period are some challenges of paracoccidioidomycosis treatment [4, 6], and difficulty in adhering to therapy is a frequent report in patient follow‐up [7, 8, 9, 10].

The objective of this study was to identify demographic and clinical characteristics and habits associated with adherence to paracoccidioidomycosis treatment. The study assessed the factors associated with adherence to paracoccidioidomycosis treatment, aiming to identify more effective approaches that reduce the aggravation of sequelae and improve patients' quality of life.

## METHODS

### Study design and participants

A cross‐sectional study including data on patients with a confirmed diagnosis of paracoccidioidomycosis who underwent drug treatment at the *Hospital Cassiano Antonio Moraes* in Vitória, state of Espírito Santo, southeastern Brazil, between 2005 and 2018. A confirmed paracoccidioidomycosis presented clinical manifestations compatible with the disease and fungal elements suggestive of *Paracoccidioides* spp. in secretions, body fluids, or lesions. This material was evaluated by direct mycological examination, double immunodiffusion, histopathological examination, or culture [4]. The outcome was the status of treatment adherence, and the exposures were demographic and clinical characteristics and alcohol/tobacco use status.

### Study variables

Sex, age (in years), and profession were chosen as demographic variables. Smoking and alcohol consumption, reported at diagnosis and discontinued during treatment, were the habits evaluated. The clinical variables were the clinical form of paracoccidioidomycosis; sites with lesions, co‐infections, and co‐morbidities; treatment drugs (amphotericin B, itraconazole, cotrimoxazole), and treatment adherence.

The acute/subacute form of paracoccidioidomycosis presented rapid evolution and dissemination of fungi in various organs and manifestations such as fever, weight loss, lymphadenomegaly, hepatosplenomegaly, digestive manifestations, skin lesions, osteoarticular involvement, and pulmonary involvement. The chronic form was insidious, with symptoms lasting for months, with possible involvement of lungs, upper aerodigestive mucosa, and skin. The mixed form had characteristics of the acute/subacute and chronic forms [4].

Patients were identified as smoking if they consumed nicotine from tobacco derivatives daily in the form of cigarettes, cigars, pipes, straw cigarettes, and hookahs. Alcohol consumption was defined as the patient consuming more than 50 g of ethanol per day [11].

Treatment adherence occurred when the patient used the medication continuously. Non‐adherence occurred in cases with discontinuous treatment, represented by an absence of itraconazole administration lasting 15 days or more or cotrimoxazole for 30 days or more [4, 10].

### Data analysis

Absolute and relative frequencies were represented as categorical variables, and Pearson chi‐squared or Fisher's exact tests were applied to compare adherence and non‐adherence to treatment groups. The medians and interquartile ranges represented continuous quantitative variables, and the comparisons between adhering and non‐adhering groups were carried out using the Mann–Whitney *U* test. The effect measures were determined using the odds ratio, with a significance level of 95%. Differences were considered statistically significant for *p*s < 0.05. Data were analyzed in SPSS® version 20 (IBM®).

### Ethical clearance

The study protocol was approved by the Ethics Committee for Research with Human Beings of the Health Sciences Center, Federal University of Espírito Santo (opinion number 3,943,304) and is in accordance with the Helsinki Declaration of 1975, as revised in 1983.

## RESULTS

The 198 patients included in the study were all diagnosed with paracoccidioidomycosis, which was confirmed by direct mycological examination (*n* = 100; 50.5%), histopathology (*n* = 106; 53.5%), or serology (*n* = 150; 75.7%). Imaging tests from different sites were performed for 182 patients (91.9%).

Most paracoccidioidomycosis cases occurred in males (93.4%), representing a ratio of 14.2 men per woman. The patients were predominantly adults (median age = 51 years), farmers (80.6%), smokers (90.4%), and alcohol consumers (84.8%). Treatment adherence was observed in 112 (56.6%) patients and was not associated with sex (*p* = 0.155), age (*p* = 0.132), profession (*p* = 0.638), or tobacco use at the time of diagnosis (*p* = 0.113). In contrast, there was a statistically significant difference in alcohol consumption before the start of treatment between the adherence and non‐adherence groups (*p* = 0.016) (Table 1).

**TABLE 1:** Characteristics of paracoccidioidomycosis cases at the diagnosis according to adherence to treatment

**Variable**	**Adherence (*N* = 112)**	**Non‐adherence (*N* = 86)**	**OR (95% CI)**	***p***	**Total (*N* = 198)**
**Sex**					
Female	10 (8.9%)	3 (3.5%)	2.712 (0.723–10.177)	0.155^a^	13 (6.6%)
Male	102 (91.1%)	83 (96.5%)			185 (93.4%)
**Age**					
Median (IR)	52 (45–58)	49 (41–58)	–	0.132**^c^**	51 (42.75–58)
**Profession** **^b^**					
Farmer	88 (78.6%)	71 (82.5%)	0.826 (0.373–1.829)		159 (80.3%)
Other^d^	18 (16.1%)	12 (14.0%)			30 (15.2%)
No information	6 (5.3%)	3 (3.5%)			9 (4.5%)
**Smoking**					
Yes	98 (87.5%)	81 (94.2%)	0.432 (0.149–1.251)	0.113**^b^**	179 (90.4%)
No	14 (12.5%)	5 (5.80%)			19 (9.6%)
**Alcohol consumption**					
Yes	89 (79.5%)	79 (91.9%)	0.343 (0.140–0.842)	0.016**^b^**	168 (84.8%)
No	23 (20.5%)	7 (8.1%)			30 (15.2%)

Abbreviations: CI = confidence interval; IR = interquartile range; OR = odds ratio.

^a^ Fisher's exact test.

^b^ Pearson's chi‐squared test.

^c^ Mann–Whitney *U* test

^d^ Others: construction workers (*n* = 11), service workers (*n* = 10), students (*n* = 6), drivers (*n* = 2), and merchants (*n* = 1).

Most patients who adhered to treatment and consumed tobacco or alcohol at diagnosis stopped smoking (54.1%) and consuming alcohol (74.2%). Meanwhile, most of those who did not adhere to treatment continued to smoke (74.1%) and consume alcohol (64.6%) during treatment. Therefore, discontinuing such habits was associated with greater adherence to therapy, with adherence three times greater among patients who stopped smoking relative to those who did not (*p* = 0.000) and five times greater among those who stopped consuming alcohol (*p* = 0.000) (Table 2).

**TABLE 2:** Discontinuation of tobacco and alcohol consumption during treatment relative to treatment adherence

**Variable**	**Adherence**	**Non‐adherence**	**OR (95% CI)**	***p***
**Discontinuation of smoking**	**(*N* = 98)** ^b^	**(*N* = 81)** ^b^		
Yes	53 (54.1%)	21 (25.9%)	3.365 (1.781–6.358)	<0.001^a^
No	45 (45.9%)	60 (74.1%)		
**Discontinuation of alcohol consumption**	**(*N* = 89)** ^c^	**(*N* = 79)** ^c^		
Yes	66 (74.2%)	28 (35.4%)	5.227 (2.697–10.129)	<0.001^a^
No	23 (25.8%)	51 (64.6%)		

Abbreviations: CI = confidence interval; OR = odds ratio.

^a^ Pearson's chi‐squared test.

^b^ Some non‐smokers were excluded at the diagnosis.

^c^ No alcohol consumers were excluded at the diagnosis.

The chronic form of the disease predominated among the patients (84.3%), with the most affected sites being the lungs (85.4%) and oropharynx/nasopharynx (57.1%). The co‐infections and co‐morbidities identified were hypertension (*n* = 29), tuberculosis (*n* = 12), HIV (*n* = 7), neoplasm (*n* = 7), diabetes (*n* = 4), and helminthiasis (*n* = 2). Most of the cases were treated with cotrimoxazole (93.3%). There were no significant differences between the adherence and non‐adherence groups for the clinical form of the disease, sites affected, and drugs used in the treatment (Tables 3 and 4).

**TABLE 3:** Clinical characteristics of paracoccidioidomycosis cases at the time of diagnosis relative to adherence to treatment

**Variable**	**Adherence (*N* = 112)**	**Non‐adherence (*N* = 86)**	**OR (95% CI)**	***p***	**Total (*N* = 198)**
**Clinical form**					
Chronic	95 (84.8%)	72 (83.7%)	1.087 (0.503–2.349)	0.833**^b^**	167 (84.3%)
Acute/mixed	17 (15.2%)	14 (16.3%)			31 (15.7%)
**Sites affected**					
Adrenal	22 (19.6%)	13 (15.1%)	1.373 (0.647–2.912)	0.408**^b^**	35 (17.7%)
Spleen	1 (0.9%)	3 (3.5%)	0.249 (0.025–2.439)	0.319^a^	4 (2.0%)
Liver	2 (1.8%)	2 (2.3%)	0.764 (0.105–5.533)	0.789^a^	4 (2.0%)
Larynx	11 (9.8%)	13 (15.1%)	0.612 (0.259–1.442)	0.258**^b^**	24 (12.1%)
Lymph nodes	22 (19.6%)	17 (19.8%)	0.992 (0.490–2.011)	0.983**^b^**	39 (19.7%)
Oro/nasopharynx	60 (53.6%)	53 (61.6%)	0.718 (0.406–1.272)	0.256**^b^**	113 (57.1%)
Bone	2 (1.8%)	3 (3.5%)	0.503 (0.082–3.079)	0.449^a^	5 (2.5%)
Skin	22 (19.6%)	14 (16.3%)	1.257 (0.601–2.630)	0.543**^b^**	36 (18.2%)
Lung	95 (84.8%)	74 (86.0%)	0.906 (0.408–2.015)	0.809**^b^**	169 (85.4%)
CNS	6 (5.4%)	8 (9.3%)	0.552 (0.184–1.655)	0.283**^b^**	14 (7.1%)
GIT	3 (2.7%)	1 (1.2%)	2.339 (0.239–22.892)	0.634^a^	4 (2.0%)
**Treatment drugs** ^b^					
Amphotericin B	21 (18.8%)	21 (24.4%)	0.714 (0.361–1.415)	0.333**^b^**	42 (21.2%)
Co‐trimoxazole	104 (92.9%)	82 (95.3%)	0.634 (0.184–2.180)	0.558**^b^**	186 (93.9%)
Itraconazole	26 (23.2%)	16 (18.6%)	1.323 (0.658–2.659)	0.432**^b^**	42 (21.2%)

Abbreviations: CI = confidence interval; CNS = central nervous system; GIT = gastrointestinal tract; OR = odds ratio.

^a^ Fisher's exact test.

^b^ Pearson's chi‐squared test.

**TABLE 4:** Drugs used in treating paracoccidioidomycosis cases

**Variable**	**Adherence** **(*N* = 112)**	**Non‐adherence** **(*N* = 86)**	**OR (95% CI)**	***p***	**Total** **(*N* = 198)**
**Treatment drugs** ^b^					
Amphotericin B	21 (18.8%)	21 (24.4%)	0.714 (0.361‐1.415)	0.333^a^	42 (21.2%)
Co‐trimoxazole	104 (92.9%)	82 (95.3%)	0.634 (0.184‐2.180)	0.558^a^	186 (93.9%)
Itraconazole	26 (23.2%)	16 (18.6%)	1.323 (0.658‐2.659)	0.432^a^	42 (21.2%)

Abbreviations: CI = confidence interval; OR = odds ratio.

^a^ Pearson's chi‐squared test.

^b^ Amphotericin B was used in acute forms (*n* = 9), and severe chronic multifocal forms (*n* = 33) and maintenance treatment was extended with co‐trimoxazole (*n* = 28) or itraconazole (*n* = 12) in subsequent months.

## DISCUSSION

This study demonstrated a high frequency of non‐adherence to treatment in cases of paracoccidioidomycosis. Smoking and frequent alcohol consumption were relevant factors associated with treatment adherence, while demographic and clinical characteristics were not. The profile of patients was similar to other settings, with middle‐aged men, farmers, smokers, and alcohol consumers being predominant [6, 10, 12].

Despite the high frequency of discontinuous treatment, adherence to therapy in the study population was higher than previously reported in Rio de Janeiro and Mato Grosso do Sul [8, 9], except in one study [10], which found 69.2% adherence. Some hypotheses were raised in previous studies to explain non‐adherence, such as the type of clinical manifestation [10], long‐term therapy, sociocultural factors, and tobacco and alcohol dependencies [8, 13].

The clinical manifestations predominant in the study population were compatible with the high prevalence of chronic paracoccidioidomycosis, characterized by frequent lung lesions and oropharynx/nasopharynx [4, 14]. Furthermore, the predominance of chronic manifestations independent of treatment adherence status in the present study is consistent with the literature, which indicates that 74%–96% of disease cases have this clinical form [4, 14]. Unsurprisingly, then, no disease manifestation was associated with therapy adherence. Nevertheless, a previous study showed that lung involvement was related to higher adherence to treatment due to the discomfort caused by the symptoms [10].

Despite a previous report of better adherence to treatment in patients using itraconazole relative to those using co‐trimoxazole [15], no differences in adherence to therapy were found in this study between medication groups, possibly due to the low number of patients using itraconazole. Itraconazole administration has a shorter duration and a more comfortable dosage (two to four capsules per day for 9 to 15 months) than cotrimoxazole (four to six capsules per day for 18–24 months), which may contribute to better adherence [15]. Nevertheless, the efficacy and payment‐free access to cotrimoxazole in the Brazilian public health system contribute to this being the most commonly used drug for paracoccidioidomycosis [16].

Demographic characteristics did not affect treatment adherence and reflected the typical epidemiological profile of the disease, which is more common in people older than 30 years [4, 14]. Besides, clinical manifestations are affected by the hormone ß‐estradiol in women, which prevents the differentiation of the fungus to its pathogenic form by modulating the cellular immune response, making men more prone to presenting signs and symptoms [6, 17]. In addition, a high proportion of farmers with the disease, similar to previous studies [6, 10, 12, 18], is related to transmission by managing contaminated soil, which leads to the inhalation of the micellar fungi [4, 19].

The consumption of tobacco and alcohol during treatment was associated with non‐adherence to therapy. Poor living conditions often correlate with tobacco and alcohol dependency, leading to inappropriate medication use and less family supervision, contributing to forgetfulness and interruption of medication use [8]. Tobacco and alcohol consumption has also been associated with an increased risk of developing chronic paracoccidioidomycosis [20]. Therefore, the results indicate that efforts to discontinue alcohol and tobacco use may improve adherence to therapy and quality of life among patients with paracoccidioidomycosis, reducing the aggravation of sequelae.

The present study has limitations inherent to the use of medical records, such as inaccurate or absent data. Furthermore, adherence was self‐reported, and due to the absence of data on serum levels of antifungals, it can be overestimated. As a tertiary hospital focused on adults, the clinical profile of patients may differ from other health centres, leading to potential underestimation in the prevalence of paracoccidioidomycosis in younger individuals and, consequently, the prevalence of the acute form of the disease.

## CONCLUSION

Paracoccidioidomycosis treatment is complex due to its long duration and patients' frequent dependency on tobacco and alcohol. The results indicate increased treatment adherence in subjects who can discontinue these chemical substances during treatment, reinforcing the relevance of offering supporting therapeutics for dependency.

## AUTHOR CONTRIBUTIONS

WLLK: conception and design of the study, aquisition of data, analysis and interpretation of data, and drafting the article. RDCB and HEO: aquisition of data, and final approval of the version to be submitted. AF, PMP, TRGV and SGT: conception and design of the study, and final approval of the version to be submitted. CRV: conception and design of the study, analysis and interpretation of data, and drafting the article.

## CONFLICT OF INTEREST

The authors declare no conflict of interest.

## Data Availability

Data are subject to third‐party restrictions.
